# Experimental Verification for Numerical Simulation of Thalamic Stimulation-Evoked Calcium-Sensitive Fluorescence and Electrophysiology with Self-Assembled Multifunctional Optrode

**DOI:** 10.3390/bios13020265

**Published:** 2023-02-13

**Authors:** Yao-Wen Liang, Ming-Liang Lai, Feng-Mao Chiu, Hsin-Yi Tseng, Yu-Chun Lo, Ssu-Ju Li, Ching-Wen Chang, Po-Chuan Chen, You-Yin Chen

**Affiliations:** 1 Department of Biomedical Engineering, National Yang Ming Chiao Tung University, Taipei 112304, Taiwan; 2 Graduate Institute of Intellectual Property, National Taipei University of Technology, Taipei 10608, Taiwan; 3 The Ph.D. Program in Medical Neuroscience, College of Medical Science and Technology, Taipei Medical University and National Health Research Institutes, Taipei 11031, Taiwan; 4 The Ph.D. Program in Medical Neuroscience, College of Medical Science and Technology, Taipei Medical University, Taipei 11031, Taiwan; 5 School of Electrical and Computer Engineering, Georgia Institute of Technology, Atlanta, GA 30332, USA

**Keywords:** fiber photometry, deep brain stimulation, optical biosensor, volume of tissue activated, Monte Carlo simulation

## Abstract

Owing to its capacity to eliminate a long-standing methodological limitation, fiber photometry can assist research gaining novel insight into neural systems. Fiber photometry can reveal artifact-free neural activity under deep brain stimulation (DBS). Although evoking neural potential with DBS is an effective method for mediating neural activity and neural function, the relationship between DBS-evoked neural Ca^2+^ change and DBS-evoked neural electrophysiology remains unknown. Therefore, in this study, a self-assembled optrode was demonstrated as a DBS stimulator and an optical biosensor capable of concurrently recording Ca^2+^ fluorescence and electrophysiological signals. Before the in vivo experiment, the volume of tissue activated (VTA) was estimated, and the simulated Ca^2+^ signals were presented using Monte Carlo (MC) simulation to approach the realistic in vivo environment. When VTA and the simulated Ca^2+^ signals were combined, the distribution of simulated Ca^2+^ fluorescence signals matched the VTA region. In addition, the in vivo experiment revealed a correlation between the local field potential (LFP) and the Ca^2+^ fluorescence signal in the evoked region, revealing the relationship between electrophysiology and the performance of neural Ca^2+^ concentration behavior. Concurrent with the VTA volume, simulated Ca^2+^ intensity, and the in vivo experiment, these data suggested that the behavior of neural electrophysiology was consistent with the phenomenon of Ca^2+^ influx to neurons.

## 1. Introduction

Neurons interact largely through different neurons that exchange neurotransmitters between dendrites and axons to deliver signals [1]. Membrane potential alternations are also transmitted to neighboring neurons or distant brain areas via electrical signals [2]. Immunohistochemistry (IHC) staining is commonly used to detect changes in specific neurotransmitters and to monitor chemical signal transmission between neurons [3]. Despite the high spatial resolution of IHC staining, identifying changes in immediate neural activity is challenging when analyzing neurotransmitter transmission between neurons, and the three-dimensional (3D) structure of the neurons may be destroyed while producing neural slice specimens.

To investigate neural activity, a neural probe is usually implanted into a specific brain area to observe the electrical signal transmission between neurons and to directly detect action potential changes in the neural membrane [4]. Although this method may not yield excellent spatial data, temporal resolution can be significantly improved [5]. Owing to advances in probe fabrication, neural probes can now detect smaller changes in membrane potential [6] and may be modified to have a reduced inflammatory response [7]. Neural electrophysiology can detect in vivo changes in neural membrane potential in response to various stimuli, including electrical stimulation [8,9] and other sensory stimuli inputs [10,11]. Electrophysiology can also reveal neural firing characteristics such as spikes (high-frequency signals released by a single neuron) [12] and local field potentials (LFP) (signals emitted by a specific neural population) [13]. Notably, LFP has been used to study various brain phenomena, including the neural basis of perception [14], attention [15], and memory [16], as well as the neural basis of various neurological and psychiatric disorders [17,18,19]. LFP is thought to reflect neuron activity that significantly contributes to the regulation of excitatory neuron activity and shaping of the brain’s overall activity patterns. Previous studies have shown that LFP comprises inhibitory and excitatory postsynaptic potentials (IPSP and EPSP, respectively). Ca^2+^ influx across the neuron cell membrane can induce EPSP, which then triggers neurotransmitter release [20]. However, during electrical stimulation, the electrical current, size, and shape of the electrodes used to deliver the current, as well as the brain’s tissue properties, may generate artifacts during electrophysiological recording, making it difficult to interpret the LFP and obscuring the underlying neural activity. Therefore, to minimize the effect of electrical stimulation artifacts on LFP, researchers have used techniques such as the template subtraction method [21], which attempts to isolate the LFP produced by the underlying neural activity from the electrical artifacts.

As previously stated, because Ca^2+^ regulates neurotransmitter release when neurons generate LFP, neurologists have concentrated on Ca^2+^ studies. The development of fluorescent Ca^2+^ indicators has accelerated Ca^2+^ research in the field of neuroscience. Bioluminescent calcium-binding photoproteins, including aequorin, were the first calcium indicators utilized for cellular Ca^2+^ signaling. Aequorin was microinjected into cells to track rapid changes in intracellular Ca^2+^ by observing changes in luminosity [22]. Although Ca^2+^ indicators can offer spatiotemporal data on neural activity, they are incapable of determining whether the Ca^2+^ fluorescence changes occur in neurons because neurons and astrocytes in the brain also cause alternations in Ca^2+^ concentration when these cells are activated [23,24], causing the fluorescent signals of Ca^2+^ indicators to coincide with the activity of neurons and other glia cells. Notably, Ca^2+^ indicators can now be expressed in a specific cell type by regulating gene promoters using gene engineering. In recent years, the green fluorescent protein calcium indicator (GCaMP) family has emerged as the most widely used genetically encoded calcium indicator (GECI) for studying Ca^2+^ signals in neurons [25]. GCaMP is typically expressed in neurons using genetic engineering techniques, such as viral vectors or transgenesis. Once expressed, GCaMP fluoresces when bound to Ca^2+^, enabling researchers to measure changes in Ca^2+^ concentration using microscopy or other imaging techniques [26]. Since GECIs have been developed, technologies that measure brain activity through alterations in Ca^2+^ fluorescence signals have advanced rapidly. 

An optrode is an emerging tool that it is used to implant in the brain to acquire Ca^2+^ fluorescent signals and simultaneously monitor neural electrical activity in the deep brain area. The current utilities of the optrodes are classified according to integrated optical fiber, waveguide, and micro-light-emitting diode (microLED), which were highlighted in opsin technologies of optogenetic modulations and fluorescence sensing. Previous studies have successfully demonstrated the combination of genetics and optics for simultaneous control and monitoring of neural activity with the implantation of the optical-fiber-based optrode [27,28]. To reduce the brain injury caused by the fiber-based optrode, an alternative to optical optrodes for light delivery is to use an integrated optical waveguide with a small cross-sectional area [29]. However, there is a comparatively large propagation loss for a small-dimension waveguide; the input power of light must be increased, resulting in more energy loss and heat damage [30,31].

To address the waveguide propagation loss and dispersion of the implantable optrode [29,32], recent studies have demonstrated the microfabrication of the optrode with an integrated microLED array [33,34], which provided optical stimuli with high extraction efficiency and spatial resolution. The microLED-based optrode was performed without an external light source and appropriate coupling system, but the requirement for a power supply and heat dissipation in tissue caused some additional problems [35,36,37]. For long-term implantation, the microLED-based optrode easily suffered from electrical leaks and short-circuits caused either by degradation in the semiconducting properties [38] or by electrochemical oxidation of the internal metal layer from the ingress of moisture, liquid, and/or ionic species [39,40].

The recent development of photometric applications in optogenetics has created an increased demand for advancing engineering tools of optrodes for fluorescence sensing in vivo. An implantable neural probe with integrated waveguide based on semiconductor technology has attracted attention because of its high sensitivity in fluorescence [41] and minimization of neuronal loss [33,34]. The technique of integrated waveguide needs to overcome its high cost, high fabrication complexity, and lower integrity with the light source with coupling issue [42,43], leading to its low popularity in the field of neuroscience. This presents an opportunity to develop a robust manufacturing process for assembling optical fibers and flexible neural probes for photometric applications. To create a cost-effective optrode for detecting fluorescence, we developed a rapid, uncomplicated, and efficiency method for assembling a polyimide-based neural probe with an optical fiber. In this way, the position of the optical fiber can be freely altered and the process of optrode assembly is simpler and lower-consuming. Furthermore, to the best of our knowledge, an optical-fiber-based multichannel optrode for in vivo testing of electrical stimulation concurrently combined with fluorescence detection and electrophysiological recording has rarely been reported.

Deep brain stimulation (DBS) has been used extensively in neuroscience research [44,45] and clinical therapy [46,47] and is an efficient method for regulating synaptic plasticity accompanied by alterations in the Ca^2+^ concentration in neurons [48,49]. To unravel the relationship between DBS-evoked LFP and DBS-evoked Ca^2+^ fluorescent activity, our self-assembled optrode capable of concurrently recording LFP and Ca^2+^ fluorescence was proposed in this study. Before the in vivo experiment, the volume of tissue activated (VTA) was estimated to verify the DBS-evoked regions. Notably, VTA is used extensively in DBS research due to its many clinical applications: it can aid in assessing the best stimulation sites [50], selecting postoperative stimulation parameters [51], and directing the presurgical planning for DBS lead-insertion surgery [52]. We also investigated the Ca^2+^ fluorescence activity under DBS. Monte Carlo (MC) simulation was used to simulate the evoked Ca^2+^ fluorescence signal [53,54]. In the field of optical modeling, MC simulation has been the gold standard and most effective technique, particularly for modelling light propagation in biological tissue [55]. To apply it to our fabricated fiber-based multichannel optrode, we modified the standard voxel-based MC simulation, and both the excitation and emission wavelengths were considered. To further verify the changes between LFP and Ca^2+^ fluorescent signals evoked by DBS, AAV-GCaMPs were transfected into the ventral posteromedial thalamic nuclei (VPM) of rats, and DBS was performed using the predetermined parameters in the VTA and MC simulation. 

## 2. Materials and Methods

### 2.1. Fabrication and Design of an Optrode

The proposed optrode comprised 16 microelectrodes and a reference electrode. The microelectrode array can be used for electrophysiological recording and electrical stimulation. Three quartz masks were designed to fabricate a probe. The first mask (Mask #1) was utilized in the internal wiring structure of a chip to implement a high-density redistribution layer (RDL) on the substrate of the microelectrode array and reference electrode, as well as its accompanying wire-bonding package metal substrate and connecting wires. The second mask (Mask #2) was used to create a three-dimensional microelectrode array, reference electrode, and metal bonding pad for wire bonding. The third mask (Mask #3) was used to form the essential features of the neural probe, such as long-axis electrodes, a tip with the appropriate angle for neural implantation, and its latter portion that carried the wire bonding.

**Figure 1:**
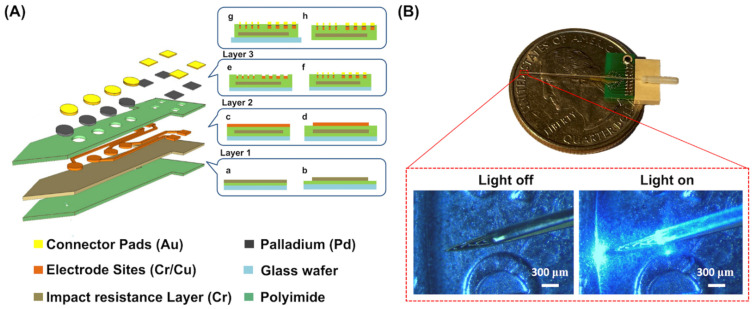
(**A**) Schematic illustration of microfabrication procedures of the optrode in the exploded-view drawing including cross-sectional and top views. The impact-resistant layer of the neural probe was made by depositing a 200 nm thick Cr layer over the polyimide to increase its strength (**a**). Wet etching was used to finish the impact-resistant layer and reserve the shape of the optrode (**b**). A second polyimide layer was vapor-deposited with a 700 nm thick Cu layer and 100 nm thick Cr layer (**c**). The 16 microelectrodes, one reference electrode, and interconnecting traces were lithographically patterned using Mask #1 (**d**). Using O_2_ plasma etching with Mask #2, windows with lithographic patterns were made on the third polyimide layer (**e**). Pd was electroformed onto the windows, and Au was electroplated on top of the Pd layer to enhance the sensing area (**f**). The three polyimide layers were lithographically patterned with Mask #3 to remove the neural probe from the glass wafer (**g**,**h**). (**B**) The photograph of our fabricated optrode assembly with the combination of optical fiber and the neural probe attached to a printed circuit board (PCB) soldered to a small strip connector. The optical fiber, which was 200 μm in diameter and 0.48 NA, was positioned to align Channel #5 of the microelectrode on the neural probe shaft. Following positioning, UV resin was spluttered on the optical fiber to fix it on the neural probe shaft. Final optrode assembly is shown on a PCB with enlarged views of light off and light off conditions.

Figure 1 shows the flow diagram for fabricating a probe with three microelectrode arrays and a reference electrode. To prepare for polyimide film removal (PI-2611, HD Microsystems, Parlin, NJ, USA), a 6-inch glass wafer was first coated with a 200 nm thick chromium (Cr) layer and a 700 nm thick Cu layer using vapor deposition. After covering, the sacrificial layer was covered with a 30 μm thick polyimide film using a spin coater and baked at 300 °C, and an impact-resistant layer was created by depositing a 200 nm thick Cr layer onto the polyimide to increase its strength. This layer was then coated with a second polyimide layer with 30 μm thickness and cured as previously mentioned. Furthermore, wet etching was performed to finish the impact-resistant layer and preserve the shape of the neural probe. The second polyimide layer was then vapor-deposited with a 700 nm thick copper (Cu) layer and 100 nm thick Cr layer. The 16 microelectrodes, one reference electrode, and interconnecting traces were lithographically patterned using Mask #1 as shown in Figure 1A(a–d).

Subsequently, the metal circuits were shielded with a third 3.2 μm thick polyimide layer, which was spun onto a trace layer. On this layer, 3.2 mm thick windows with lithographic patterns were made using O_2_ plasma etching with Mask #2 (Figure 1A(e)). To avoid cracks in the microelectrodes and bonding pads of the neural probe caused by the Kirkendall effect at the interface between gold and copper [56,57,58], which limits the utility of gold-copper coatings in high-temperature environments and when a long lifetime is sought, palladium was electroplated on the copper, which was used to suppress the voids formed at the boundary interface in the gold–copper coatings [58,59,60]. Following palladium electroplating, the process of gold plating was performed to form 3D-structured microelectrodes and bonding pads (Figure 1A(f)). To remove the optrode from the glass wafer, the three polyimide layers were lithographically patterned with Mask #3, which was etched with O_2_ plasma, and the Cu-based sacrificial layers were removed with a metal etchant as shown in Figure 1A(g,h).

After aligning the tip of an optical fiber (200 μm of diameters, 0.48 NA, Inper, Hangzhou, China) with Channel #5 of the neural probe and affixing it using UV resin (NOA65, Norland Products Inc., East Windsor, NJ, USA), the optrode was completed as shown in Figure 1B.

### 2.2. Computational Modeling of the VTA in Thalamic DBS

A 3D finite element method (FEM) model of the optrode was constructed using a commercial software package (Maxwell^®^, ANSYS, Inc., Canonsburg, PA, USA) to assess the effects of DBS. A 2 × 2 × 2-mm^3^ cube of homogeneous and isotropic brain tissue was modeled as an axisymmetric volume conductor surrounding the DBS microelectrodes. The electrical resistivities of the gold microelectrode and polyimide substrate of our self-assembled optrode were 2.439 × 10^−8^ and 1.667 × 10^16^ μm, respectively. To simulate the actual environment of the brain tissue in vivo, in vivo impedance was measured [61] using a pair of microelectrodes (Channels #1 and #4) on our self-assembled optrode with a sinusoidal voltage source (20 mV, <150 nA, at 1 kHz) generated with an LCR meter (Model: 4263B, Agilent Technologies Inc., Santa Clara, CA, USA). Subsequently, the corresponding in vivo conductivity was measured using Equation (1):

(1) $$\mathrm{Conductivity}\;\left(K\right)[S/m]=\frac{1}{\mathrm{in}\;\mathrm{vivo}\;\mathrm{resistance}(R)}\;\times \;\frac{\mathrm{distance}(D)}{\mathrm{area}(S)}$$

where **R** [Ω] was measured between Channel #1 and #4, **D** between both Channels was 450 μm, and **S**, with a microelectrode radius of 8 μm, was 200.960 μm^2^. **R** was 0.543 ± 0.005 MΩ, and **K** was calculated to be 4.120 ± 0.041 S/m. **K** was then introduced to Equation (2) to describe the current density and electric field:

(2) $$\vec{J\;}=K\vec{E}=-K\nabla V_{e}$$

where $\vec{J}$ is the current density, $\vec{E}$ is the electric field, and $V_{e}$ is the negative gradient of potential.

With a point source of current, **I_v_**, which was assumed to be infinite, $\vec{J}$ could be described with divergence properties and presented as Equation (3):

(3) $$\nabla \cdot \vec{J}{=I}_{v}=-K\nabla {\;V}_{e}$$

Under a homogeneous condition, Poisson’s equation for $V_{e}$ is described as Equation (4):

(4) $$\nabla^{2}V_{e}=-\frac{I_{v}}{K}$$

Owing to this homogeneous condition, the conservation of the current requires that **∇·**$\vec{J}$ **= 0**. Simultaneously, $V_{e}$ must satisfy a partial differential equation called the Laplace equation which presented as Equation (5):

(5) $$\nabla^{2}V_{e}=0$$

A solution for $V_{e}$ in Poisson’s equation could be described as Equation (6):

(6) $$V_{e}=\frac{1}{4\pi \varepsilon }\int \frac{\sigma dV}{r}$$

where **ε** is the dielectric permittivity, **σ** is the permittivity, and **r** is the distance between two points in a tissue. Based on these equations, the distribution of $V_{e}$ could be calculated. In this FEM model, the mesh size was set to 10 μm, the in vivo conductivity, **K**, was set to 4.120 S/m for a homogeneous and isotropic tissue medium, as mentioned earlier, and the conductivities of the gold microelectrode and polyimide substrate of the self-assembled optrode were set to 4.100 × 10^7^ and 5.998 × 10^−17^ S/m, respectively. **σ** was set to 80 in the homogeneous and isotropic tissue medium.

In this study, VTA was estimated using an activating function, defined as the second spatial derivative of an extracellular voltage along an axon [62], which can be described as Equation (7):

(7) $$f\left(n\right)=\frac{\Delta^{2}V_{e}}{\Delta x^{2}}=\frac{\left[V_{e}\left(n+1\right){-V}_{e}\left(n\right)\right]-\left[V_{e}\left(n\right){-V}_{e}\left(n-1\right)\right]}{L^{2}}=\frac{V_{e}\left(n-1\right){-2V}_{e}\left(n\right){+V}_{e}\left(n+1\right)}{L^{2}}$$

where **n** is the position of the homogeneous and isotropic tissue medium, and **L** is the grid spacing conducted in the FEM model, which is 10 μm. The regions were assumed to be activated when **f(n)** > 0.

### 2.3. Simulation of the Thalamic-DBS-Evoked Calcium Signal

The purpose of MC simulation is mainly to investigate the changes in Ca^2+^ fluorescence emission and intensities from the VTA volume in response to varying DBS intensities. Our MC simulation was performed using a lab-designed MATLAB^®^ software (2020R MathWorks Inc., Natick, MA, USA) and C code. A single simulation was conducted concurrently with 465 nm excitation light and 525 nm emitted fluorescence. The voxel size was set to 10 μm according to the mesh size from the FEM model as described in Section 2.2. Our MC simulation model was made as the optical fiber (200 μm in diameter, 0.48 NA) inserted in a 2 × 2 × 2 mm^3^ homogeneous brain tissue [63]. To estimate the photon trajectory stimulated by the fiber, the light source of the optical fiber was assumed to be a defocused, uniformly dispersed beam. The position of the light source was located at Channel #5 of the optrode, corresponding to the position in the FEM model. The launch angle was 20.5° based on the NA of the optical fiber. Furthermore, the absorption (**μ_a_**) and scattering (**μ_s_**) coefficients of the white matter were measured using an integrated sphere optical system (Ophir IS6, Ophir Optronics Solutions Ltd., Jerusalem, Israel) under both 465 nm and 525 nm laser wavelengths in order to fit the MC simulation environment to real brain conditions.

To determine the fluorescence signal profile of a single fiber implanted in a tissue, MC simulations of 10 M photon packets were emitted from the fiber, with the initial energy for each photon set to 1 weight (W). The starting coordinates and initial photon direction were selected based on the position of the optical fiber. As an optical fiber light source, a defocused, uniformly distributed beam was employed to predict the photon exit trajectories. The step size (**ΔS**) of a photon after it leaves the fiber must be less than the mean free path length of a photon in tissues; this is the reciprocal of the total attenuation coefficient. A function of random variable (**ξ**) was used to efficiently generate different step sizes for each photon step, as shown in Equation (8):

(8) $$\Delta S=\frac{-\ln\xi }{\mu_{a}{+\mu }_{s}}$$

where **μ_a_** and **μ_s_** were 4.642 cm^−1^ and 257.631 cm^−1^ for a 465 nm excitation light and 4.873 cm^−1^ and 224.074 cm^−1^ for a 525 nm emission fluorescence, respectively. 

After each propagation step, a fraction of the photon packet was absorbed. The fraction of the absorbed photon weight **ΔW** was calculated using Equation (9):

(9) $$\Delta W=(\frac{\mu_{a}}{\mu_{a}{+\mu }_{s}})W$$

The updated weight (**W′**) representing the fraction of the scattered packet was given by Equation (10):

(10) $$W^{\prime }=W-\Delta W$$

The fluorescence quantum yield (**QY**) of a fluorophore is the fraction of absorbed photons resulting in fluorescence emission. Therefore, fluorescent photon emission occurs when an excitation photon propagates into a voxel where the fluorescence **QY** exceeds 0. When the absorbed photon weight was **ΔW**, the initial weight of the fluorescent photon packet **W_f_** was calculated using Equation (11):

(11) $$W_{f}=\Delta W\;\times \;\mathrm{QY}$$

As a result, **QY** was set to 1 if the activated region exhibited normal Ca^2+^ indictor expression. Finally, the energy of emission fluorescence was denoted as **φ** and could be calculated by Equation (12):

(12) $$\varphi =\frac{W}{{\mathrm{cm}}^{2}}{=W}_{F}\times \frac{1}{\mu_{a}V}$$

where **μ_a_** is the absorption coefficient of the emission light, and **V** is the voxel size of 10^−3^ × 10^−3^ × 10^−3^ cm^3^. 

### 2.4. Setup of Fiber Photometry System

**Figure 2:**
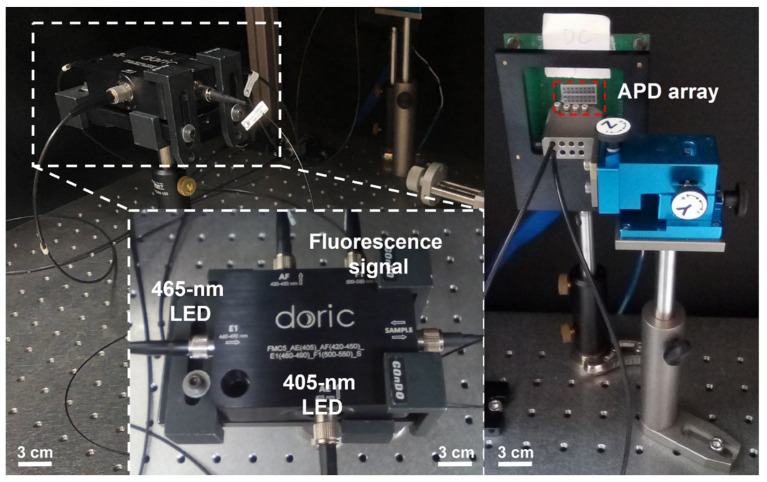
Experimental setup of the photometry system. The sources of 465 nm and 405 nm LED were collimated with dichroic mirrors to collinearly orient them into an optical fiber. The evoked fluorescence signals were collected with the same fiber. The evoked Ca^2+^-dependent and Ca^2+^-independent signals were filtered with an emission filter between 500 and 550 nm and between 420 and 450 nm, respectively. These signals were recorded at Channel #9 and Channel #25 on the APD array, respectively.

A fluorescence minicube (FMC5, Doric, Québec, Canada) was used as the light path of the photometry system. A 405 nm violet (CLED_405, Doric, Québec, Canada) and 465 nm blue LED (CLED_465, Doric, Québec, Canada) were the light sources of the system. The lens collimated the two sources into dichroic mirrors such that the sources were collinearly oriented into an optical fiber for lighting. Then, the light from these two sources traveled through the optical fiber separately. The 465 nm blue LED was used to activate GCaMP6s, and the evoked Ca^2+^-dependent signals were measured in the 500–550 nm spectral window. The 405 nm violet LED was used to evoke Ca^2+^-independent signals, which were autofluorescence in brain tissue [64] and were measured in the 420–450 nm spectral window. Two optical fibers were connected to an avalanche photodiode array (APD) (S8550-02, Hamamatsu Photonics, Hamamatsu, Japan) to limit light decay and to explore the fluorescence signals, as shown in Figure 2. The signals of GCaMP6s and autofluorescence were recorded at Channel #9 and #25 on the APD array, respectively. The fluorescence signals were transmitted using a multichannel data acquisition system (PhotoniQ Model IQSP480, Vertilon Corp., Westford, MA, USA) for further analysis.

### 2.5. Animal Preparation and Surgery 

To validate the relationship between the evoked LFP and evoked Ca^2+^ fluorescence, in vivo tests were conducted on 8-week-old Sprague–Dawley (SD) adult rats (*N* = 5) weighting 250–350 g. The rats were housed and fed ad libitum in an animal facility (12:12 light/dark cycle; light on at 7 a.m.; 20 ± 3 °C). All animal experimental designs and procedures were reviewed and approved by the Institutional Animal Care and Use Committee of the Taipei Medical University (IACUC Approval number: LAC-2020-0210), and the rats were handled following the accepted standards and regulations.

GCaMP6s was the Ca^2+^ indicator in the in vivo experiment, which was obtained from Douglas Kim and GENIE Project (Addgene plasmid # 100843; RRID: Addgene_100843). The rats received 0.25 μL of GCaMP6s virus which was injected at a rate of 0.050 μL/min for 5 min into the right ventral posteromedial thalamic nuclei (VPM) (AP: −3.48 mm, ML: 2.70 mm, DV: −6.80 mm) while rats were under isoflurane anesthesia (induction 4%; maintenance 1.5%). Two weeks after viral injection, the self-assembled optrode was implanted into the rat thalamic VPM nuclei (AP: −3.48 mm, ML: 2.50 mm, DV: −6.80 mm) under the same procedure of isoflurane anesthesia. The whole skull was coated with dental cement to strengthen its attachment to the optrode. When the optrode was firmly fixed to the skull, the holder could be released, allowing the scalp to be stitched over the dental cement mound [65].

### 2.6. Thalamic-DBS-Induced Neuronal Activity Recording: Ca^2+^ Fluorescence Signals and Electrophysiology Recordings

Under isoflurane anesthesia (induction 4%; maintenance 1.5%), the rats were mounted on a stereotaxic device, and acute fluorescence and LFP were concurrently recorded for 40 s. The first 10 s of Ca^2+^ fluorescence signals were recorded to calculate the baseline. DBS was triggered with an isolated pulse stimulator (S48, Grass Technologies, West Warwick, RI, USA), providing stimulation pulses of varying current strengths with a 0.4 ms duration, 3 Hz of frequency, and different DBS intensities (50 μA, 100 μA, 200 μA, and 300 μA) at 10–30 s during the recording. To determine the maximum DBS intensity, the total electrical energy delivered to the tissue (TEED) was used to calculate the amount of energy transferred by DBS intensity to the brain tissue using Equation (13) [66]:

(13) $$E_{\mathrm{DBS}}=\frac{{\left(I\times R\right)}^{2}\times \mathrm{pw}\times f}{R}\;(1\;s)$$

where $E_{DBS}$ is the electrical energy delivered within 1 s; **f** = frequency of 3 [Hz]; **I** = current [A]; **pw** = pulse width of 0.4 × 10^−3^ [s]; and **R** = in vivo impedance of 5.43 × 10^5^ [Ω] between Channel #1 and #4 on the neural probe. In this study, the maximum TEED under the DBS intensity of 300 μA was 5.864 $\times \;10^{-5}$ J, and the 20 s DBS was applied; therefore, the energy received by tissue was 1.172 × $10^{-3}$ J. According to Deep Brain Stimulation Management [67], when considering the safety concerns for DBS, the upper limit for charge capacity is 30 μC/cm^2^, which, converted to energy, is 1.12 × $10^{-2}$ J. To ensure the biosafety, we considered the DBS intensity of 300 μA as a proper upper limit for the DBS in this study. Based on our previous study [68], the lowest DBS intensity of 50 μA could induced stable neural responses. In addition, the increase in the DBS-evoked neural responses with the linear fashion were found by gradually increasing the stimulus intensities of 50 μA, 100 μA, and 200 μA. DBS-induced Ca^2+^ fluorescence intensities were collected using our optical-fiber-based optrode. The total output power of the optical fiber tip was adjusted to 0.2 mW. The last 10 sec was the rest of the DBS.

**Figure 3:**
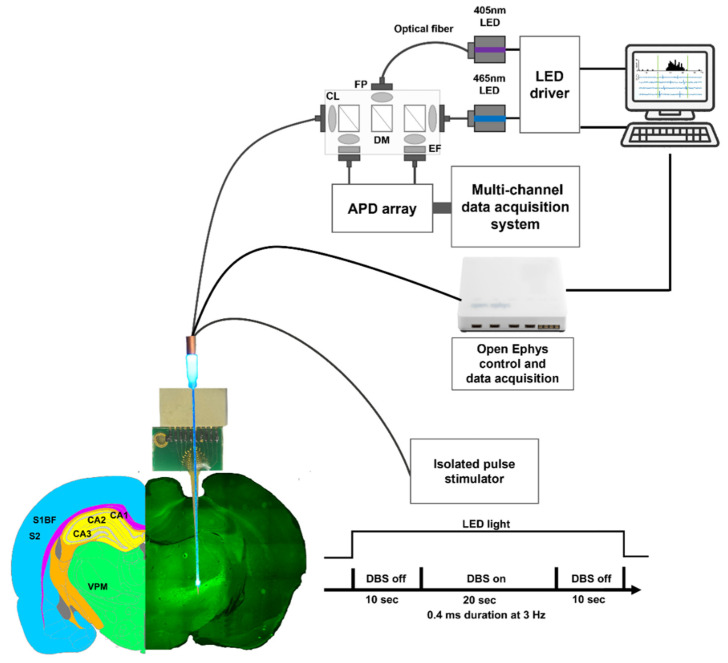
An overview of the system instrumentation for the in vivo experiment. Implantation was performed at the VPM (AP: −3.48 mm, ML: 2.70 mm, DV: −6.80 mm). Then, the optrode was linked to the photometry system, Open Ephys, and stimulator. The photometry system was attached to the fiber on the optrode. Open Ephys was attached to Channel #2, whereas the stimulator was attached to Channel #1 as the current source and Channel #4 as the reference. DBS was triggered at 10–30 s during the recordings, and the parameters of electrical stimulation were 0.4 ms at 3 Hz under different intensities (50 μA, 100 μA, 200 μA, and 300 μA). Abbreviation: CL, coupling lens; DM, dichroic mirror; EF, emission filter; FP, fiber port.

Electrophysiological recordings were also performed simultaneously using a multichannel acquisition processor (Open Ephys [69]). Neuronal LFP activity was sampled at 1 kHz and digitally filtered with a bandpass filter at 0.3–300 Hz. A graphical user interface controlled with LabVIEW (LabVIEW 2017, National Instruments, Austin, TX, USA) served as the main controller for the entire data acquisition system. Offline data were retrieved using the LabVIEW interface. Figure 3 shows the in vivo experimental setup. After electrical stimulation, the rats were sacrificed, and their brains were extracted to confirm GCaMP6s expression. This was conducted to ensure the transfection of the adeno-associated virus (AAV). Owing to the presence of a green fluorescent protein (GFP) gene segment located in the plasmid transported by the AAV, infected neurons could be observed using a fluorescent microscope (BX61, Olympus, Tokyo, Japan) at a 475 nm excitation wavelength.

### 2.7. Data Analysis

To create an averaged evoked LFP, its amplitudes were clipped every 333 ms over the 15–25 s recording period in response to fluorescence signal emergence. Then, an absolute value of the evoked response amplitudes at 30 ms post-stimulus (denoted as ∑LFP) was calculated by summing the averaged evoked LFP. ∑LFP changes were used to evaluate the stability of the evoked responses induced by the thalamic stimulation.

The raw Ca^2+^ fluorescence intensity was mixed with a high-frequency noise caused by the photometric recording instrument, which should be filtered out with a 100-Hz low-pass filter. The change in Ca^2+^ concentration is expressed as ΔF/F = (F_s_ − F_0_)/F_0_, where F_s_ is the fluorescence intensity during electrical stimulation, and F_0_ is the mean fluorescence intensity before stimulation. The ΔF/F ratio was then averaged over the 15–25 s recording period because fluorescence signals increased about 5 s later [70]. Both LFP and Ca^2+^ fluorescence intensities were analyzed using MATLAB^®^. 

### 2.8. Statistical Analysis

To verify the relationship between LFP and Ca^2+^ fluorescence intensity in vivo, a linear data fit with a corresponding coefficient of determination (***R*^2^**) was used to determine the relationships between VTA volume and simulated Ca^2+^ fluorescence intensities, VTA volume and ∑LFP, simulated Ca^2+^ fluorescence intensities and Ca^2+^ fluorescence intensity in vivo, and ∑LFP and Ca^2+^ fluorescence intensity in vivo. The higher the ***R*^2^** value, the more sensitive the positive to the paired items under stimulus intensities. The linear curve fitting was conducted with SPSS version 26.0 (SPSS Inc., Chicago, IL, USA). The significance level was set at *p* < 0.05. The results were expressed with mean values and standard error of the mean (mean ± SEM).

## 3. Results

### 3.1. Estimation of VTA Volume

**Figure 4:**
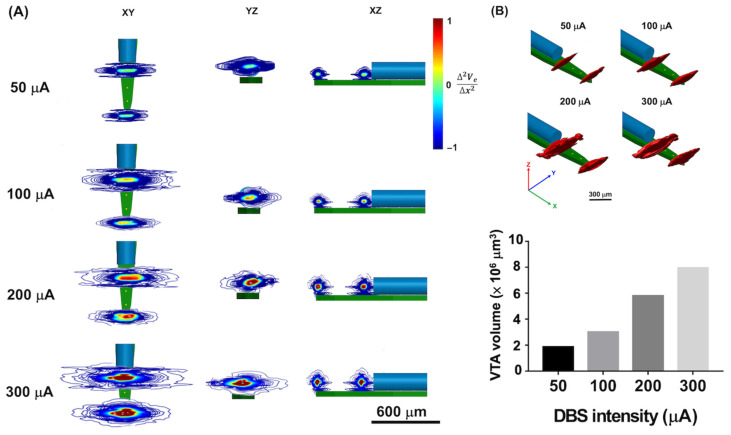
The stimulus effects under different DBS intensities. (**A**) The two-dimensional view of the electrical effects under different stimulus intensities at the range of *f*(*n*) *=* −1~1. The effects of thalamic stimulation could be investigated under different DBS intensities (50 μA, 100 μA, 200 μA, and 300 μA). With the higher DBS intensities, a larger influenced region could be observed. (**B**) The value of voxels with *f*(*n*) > 0 were estimated as the VTA volume under different DBS intensities at the *K* = 4.120 S/m. The stronger the DBS intensity, the larger the VTA volume observed.

For thalamic DBS from the optrode, the effects of all four different DBS intensities (50 μA, 100 μA, 200 μA, and 300 μA) with comparable spatial patterns of activation in the XY, YZ, and XZ planes are shown in Figure 4A. Stimulus current density modulated the effects of DBS from the optrode, and the affected regions were observed to concentrate on Channels #1 and #4 (current source and reference, respectively). Stronger effects of the stimulus current density were also observed to concentrate on the microelectrodes of current source and reference.

Subsequently, the VTA volume was estimated when the value exceeded 0, which represented the activated area based on **f(n)** (Equation (7)). Increasing the stimulus current density resulted in a larger VTA volume (Figure 4B). Because the optrode was simulated in the same homogeneous brain tissue, the simulation results suggest that a change in stimulus current density could mediate a change in VTA volume.

### 3.2. Estimation of Simulated Ca^2+^ Fluorescence Intensity

**Figure 5:**
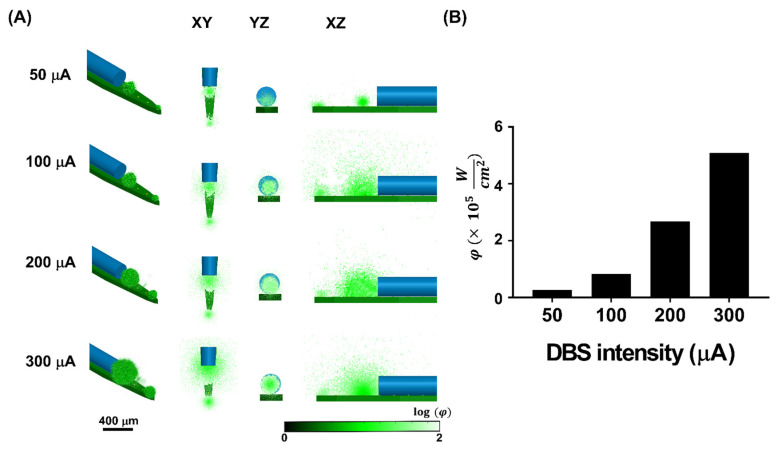
(**A**) The two-dimensional view of fluorescence distribution under different stimulus intensities. The stronger the DBS intensity, the larger the emitted fluorescence distribution observed, corresponding to the tendency of the VTA. (**B**) The estimation of fluorescence intensities in the voxels of the 3D MC model was calculated as **φ** ($\frac{W}{{cm}^{2}}$). The stronger fluorescence intensity was also performed with the higher DBS intensity.

Figure 5A shows the fluorescence distributions evoked by thalamic DBS. The activated regions were derived from VTA, and **QY** was 1 in these voxels of regions assuming that GCaMPs were fully expressed in the activated regions. The simulated Ca^2+^ fluorescence intensity was investigated by concentrating on the current source and the reference. The stronger simulated Ca^2+^ fluorescence intensity also corresponded to the stronger DBS intensity. To validate the value of simulated Ca^2+^ fluorescence, **φ** in every voxel was summed up in response to different DBS intensities (Figure 5B). A larger **φ** was found under stronger DBS intensity. In the MC simulation, because the activated regions were predetermined by the VTA volume, the similar distribution of VTA volume and Ca^2+^ fluorescence signal could be investigated.

### 3.3. Acute Ca^2+^ Fluorescence and LFP Recordings under In Vivo Thalamic Stimulation 

**Figure 6:**
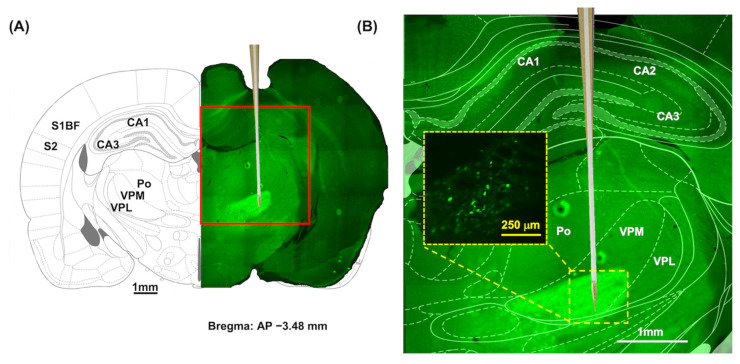
The expression of GCaMP6s in VPM. (**A**) The coronal brain tissue slice at the AP level of −3.48 mm relative to the Bregma. Abbreviations: AP, anteroposterior; Po, the posterior thalamus nuclear group; S1BF, barrel field of primary somatosensory cortex; S2, secondary somatosensory cortex; VPL, ventral posterolateral thalamic nucleus; VPM, ventral posteromedial thalamus nucleus. (**B**) An enlarged fluorescent image of the yellow rectangle shows the significant GcaMP6s–GFP expression in the thalamic VB complex, including VPM nuclei. Wide-field image of the enlarged thalamic region (yellow dotted box) shows thalamocortical relay neurons labeled with GcaMP6s–GFP.

Figure 6A shows the GCaMP expression and optrode position. The optrode was implanted at an anteroposterior (AP) level of −3.48 mm relative to the Bregma. Using the rat brain atlas, the position of the optrode was determined to be VPM (AP: −3.48 mm, ML: 2.50 mm, DV: −6.80 mm). Figure 6B shows an enlarged fluorescent image. The expression of GCaMP6s–GFP was investigated with the fluorescence microscope using the excitation of 475 nm wavelength light. 

**Figure 7:**
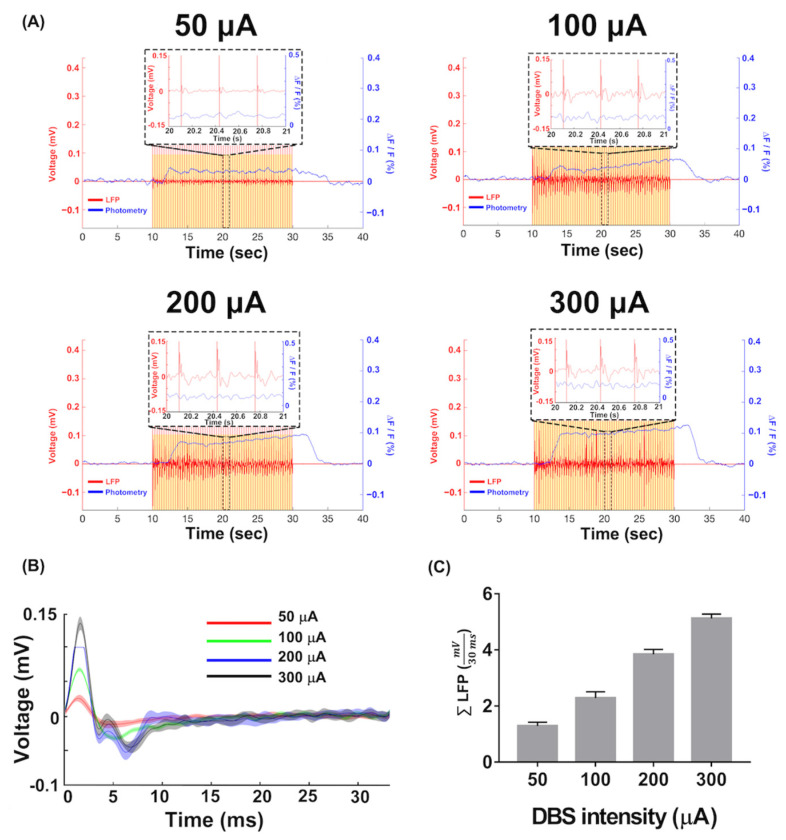
The acute Ca^2+^ fluorescence and LFP recordings in vivo. (**A**) The acute Ca^2+^ fluorescence and LFP simultaneously recorded from the right VPM electrical stimulation using the self-assembly optrode. Different DBS intensities were applied in the experiment. Acute reaction of LFP could be investigated at the time point of thalamic stimulation, while the reaction of the Ca^2+^ signal was about 5 sec later than thalamic stimulation. (**B**) The average of the amplitudes from 30 ms of evoked LFP clipped every 333 ms under different DBS intensities. Higher amplitude could be observed with higher DBS intensities. The lines indicated the mean of the LFP amplitude and shaded areas indicated the SEM. (**C**) The ∑LFP, which is the absolute value of the evoked response amplitudes at 30 ms post-stimulus under different DBS intensities. Corresponding to the result before, higher values also could be seen with higher DBS intensities. The ∑LFP is presented as mean ± SEM.

Acute Ca^2+^ fluorescence and LFPs were recorded while the rats were exposed to varying DBS intensities. Figure 7A shows the in vivo recordings of acute Ca^2+^ fluorescence and the LFP. The first 10 sec of recording indicated the Ca^2+^ fluorescence baseline, whereas the last 10 sec indicated stimulation rest. The DBS was conducted every 10 s during the recording duration. Stimulation artifacts were observed in the LFP recording, corresponding to the DBS frequency. The increased DBS intensity enabled the observation of an increase in both acute Ca^2+^ fluorescence and LFP. In addition, increased Ca^2+^ fluorescence signals were observed rising about 5 s after DBS initiation. Increasing the DBS current density induced a stronger response in both LFP and Ca^2+^ fluorescence signals, consistent with the simulated results in VTA volume and MC simulation. Figure 7B shows the first 30 ms of the average amplitudes from the elicited LFP measurements taken every 333 ms. Similar LFP amplitude patterns were observable, even when the DBS intensity was varied. Subsequently, the first 30 ms of average amplitudes under different DBS intensities were summed up with the absolute value to quantify the effects of varying DBS intensities and denoted as ∑LFP, as shown in Figure 7C. To determine the relationship between DBS-evoked LFP and DBS-evoked Ca^2+^ fluorescence signals under varying stimulus current densities, ∑LFP was further verified using VTA volume and simulated Ca^2+^ fluorescence signals with linear curve fitting.

### 3.4. Linear Relationship among VTA Volume, Simulated Ca^2+^ Fluorescence Intensity, Ca^2+^ Fluorescence Intensity In Vivo, and ∑LFP

**Figure 8:**
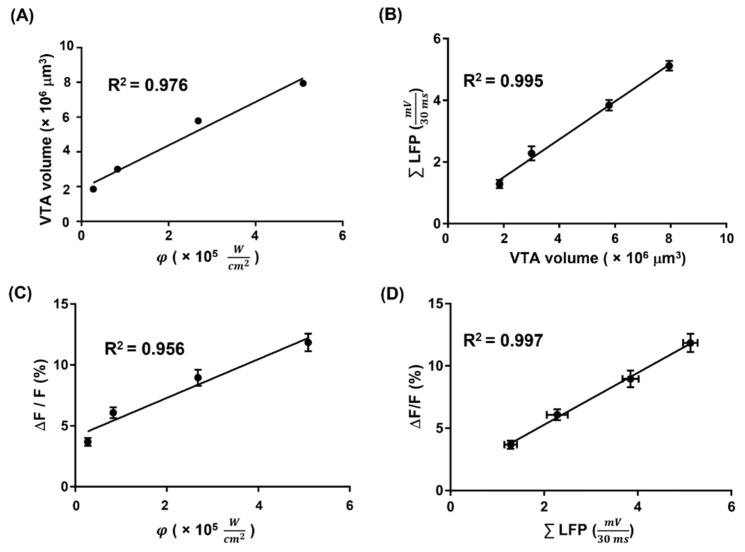
The comparison of VTA volume, simulated Ca^2+^ fluorescence intensity, Ca^2+^ fluorescence intensity in vivo, and ∑LFP (**A**) The comparison between VTA volume and simulated Ca^2+^ fluorescence intensity, ***R*^2^** = 0.976 (*p* = 0.012). (**B**) The comparison between VTA volume and ∑LFP, ***R^2^*** = 0.995 (*p* = 0.002). The ∑LFP is presented as mean ± SEM along the *Y*-axis. (**C**) The comparison between simulated Ca^2+^ fluorescence intensity and Ca^2+^ fluorescence intensity in vivo, ***R*^2^** = 0.956 (*p* = 0.022). The Ca^2+^ fluorescence intensity in vivo is presented as mean ± SEM along the *Y*-axis. (**D**) The comparison between Ca^2+^ fluorescence intensity in vivo and ∑LFP, ***R*^2^** = 0.997 (*p* = 0.001). The ∑LFP and the Ca^2+^ fluorescence intensity in vivo are presented as mean ± SEM along the *X*-axis and *Y*-axis, respectively.

Figure 8A shows the linear curve fitting between the VTA volume and simulated Ca^2+^ fluorescence intensity. The coefficient of determination (***R*^2^**) between the VTA volume and simulated Ca^2+^ fluorescence intensity was 0.976. The significant linearity indicated that the two experiments produced similar results. Figure 8B shows the linear curve fitting between ∑LFP and VTA volume for a tissue conductivity of 4.120 S/m. An optimal fit (***R*^2^** = 0.995) between ∑LFP and VTA volume was obtained. 

Figure 8C depicts the correlation between the simulated Ca^2+^ fluorescence intensity and the Ca^2+^ fluorescence intensity in vivo. The optimal coefficient of determination (***R*^2^** = 0.956) between simulated Ca^2+^ fluorescence intensity and Ca^2+^ fluorescence intensity in vivo was obtained for a **QY** of 1. Figure 8D shows the linear curve fitting between ∑LFP and Ca^2+^ fluorescence intensity in vivo. The optimal coefficient of determination (***R*^2^** = 0.997) between ∑LFP and Ca^2+^ fluorescence intensity in vivo was obtained. This result indicated an ideal sensitivity to predict the evoked Ca^2+^ fluorescence intensity in vivo based on the evoked LFP. Therefore, significant linear relationships existed among the VTA volume, simulated Ca^2+^ fluorescence intensity, Ca^2+^ fluorescence intensity in vivo, and ∑LFP.

## 4. Discussion

### 4.1. The Advance of Self-Assembled Optrode

In this study, our self-assemble optrode was capable of concurrently recording Ca^2+^ fluorescence signals and electrophysiology. The optical fiber was aligned with Channel #5 of the optrode, whereas Channel #1, #2, and #4 served as the current source, recording site, and reference, respectively. In addition, UV resin was employed as the adhesive owing to its transparency and ability to solidify when exposed to UV light. Its former characteristics minimized the light absorption loss, whereas its latter characteristics facilitated the optrode-assembly process.

Optrode assembly has been demonstrated in previous studies. Stocke and Samuelsen used a 3D-printed self-made fixture to firmly attach an optical fiber to the optrode [71]. The design of the self-made fixture could be modified to accommodate various diameters and lengths of optical fibers, and the firmness of the 3D-printed self-made fixture was shown to withstand long-term in vivo recording (12 days). Therefore, a 3D-printed self-made fixture that complies with the probe and optical fiber specifications may be an improved method for chronic in vivo recording.

Sileo, et al. [72] adhered a tapered optical fiber to a multichannel probe using UV resin. The tapered optical fiber provided homogenous illumination on the recording site, thereby reducing the loss of photonic energy by suppressing photon propagation in the brain tissue. Although the tapered optical fiber was demonstrated to be less invasive in the in vivo experiment [73], the tapered-optical-fiber-based optrode could only capture the electrophysiological signal evoked by the excitation light. Unlike the tapered-optical-fiber-based optrode, our self-assembled optrode could simultaneously record fluorescence and electrophysiological signals evoked by electrical stimulation. In addition, the microelectrode array of the optrode enabled accurate electrical stimulation and recording of small brain regions, indicating that the self-assembled optrode is a promising biosensor for investigating acute neuronal activity in vivo under varying DBS intensities.

### 4.2. The Correlation between Simulated Results and In Vivo Experiments 

Due to the assumed normal expression of GCaMPs in the VTA regions, the linear correlation data showed a strong positive association between VTA and simulated Ca^2+^ fluorescence intensity. The **QY** of the VTA region was assumed to be 1, so simulated fluroescence would be emitted once the region was activated by the excitation light, indicating that the VTA volume had a strong positive correlation with the simulated Ca^2+^ fluorescence.

The most remarkable finding was that the VTA volume corresponded to the ∑LFP under varying DBS intensities, indicating that the predicted VTA volume was significantly correlated with the summation of the LFP amplitude. VTA was judged to be the most important benchmark for measuring the effects of the stimulation [74], whereas LFP was the neuronal response generated by different DBS parameters [75]. The present study effectively connected the vital simulation to the in vivo state by demonstrating that changes in VTA volume maintained a significant positive correlation with varying DBS intensities, indicating that VTA volume estimation using FEM with a homogeneous and isotropic medium may be appropriate in the rodent model. 

However, the correlation between the simulated Ca^2+^ fluorescence intensity and the Ca^2+^ fluorescence intensity in vivo was weak, probably because **QY** was set to 1 in the MC simulation, which did not reflect the real condition of GCaMP expression in the activated regions and led to a bias in the MC simulation model compared to the in vivo experiment. Based on the previous studies, the efficiency of GCaMP expression should be considered in order to simulate the real conditions of an animal model. For instance, the GCaMP expression ratio varied at distinct post-injection time points [76]. Because several factors such as transgenic animal [77], different strains of AAV-mediated expression [78], and the plasmid design affect the ratio of efficient GCaMP expression [25,79], the **QY** of the emitted fluorescence should be dynamically adjusted for the different factors in order to satisfy the necessary conditions for MC simulation.

The linear curve fitting results showed that both VTA volume and simulated Ca^2+^ fluorescence significantly and positively correlated with in vivo signals under varying DBS intensities, although the MC simulation at this stage could not match the actual condition of the animal model. Therefore, unnecessary in vivo experiments and optrode fabrication can be reduced by incorporating VTA and MC simulation into the optrode design phase.

### 4.3. Comparison of the Ca^2+^ Photometry and Electrophysiology

The linear curve fitting results indicated that Ca^2+^ fluorescence intensity in vivo correlated with LFP under varying DBS intensities, indicating a link between Ca^2+^ and neural activity. Without a doubt, calcium facilitates the neuronal transmission of neurotransmitters and the subsequent generation of action potentials [80,81].Therefore, investigating alterations in the Ca^2+^ signal in brain tissue is an appropriate method for confirming neural activity. 

Ca^2+^ photometry has gained widespread application in neurology [26,82,83] owing to its ability to provide recordings free of electrical artifacts. Electrophysiological investigations, notably those aiming to understand how DBS affects the neurons in the target structure, have met major challenges owing to artifacts; hence, several strategies for artifact removal have been developed [84]. However, unlike electrophysiology, the photometry method for measuring calcium indicators suffers from poor temporal resolution. GCaMP calcium measurement provides an integrated signal due to many spikes, whereas conventional single-unit electrophysiology detects individual action potentials [85]. Furthermore, the decay time and the rise time of GCaMPs exceeded the electrical response and the electrical stimulation periods in this study [85], making it difficult to observe neural activity during the electrical stimulation period. Fiber photometry has additional drawbacks because it further averages signals across a population of neurons and from several neuronal compartments (dendrites and soma). A rise in fiber photometry signals might suggest either an increase in overall firing or an increase in population synchronization. Recent research in the striatum comparing neuronal firing and fiber photometry (using simultaneous multi-unit electrophysiology and fiber photometry) revealed that although fiber photometry signals were longer and may represent dendritic Ca^2+^ influx related to back propagation, an initial phase of calcium signal correlated well with firing [86], suggesting that Ca^2+^ comprises neuronal firing and the dendritic Ca^2+^ influx phenomenon.

Despite the limitation of the fiber photometry technique, the Ca^2+^ was still an important biomarker for observing neural reaction, due to its function for mediating the release of neural transmitters. Especially in some disease models, such as Parkinson’s disease [44], Alzheimer’s disease [45], and depression [87], fluorescence signals of Ca^2+^ have helped to reveal abnormal brain circuits and networking. With the development of the photometry technique and genic engineering, the limitations of measurement and the Ca^2+^ indicators may be overcome in the future.

## 5. Conclusions

In this study, the optrode successfully was fabricated with an assembly process for a low-cost, simple, and spatial flexibility of optical fiber onto the microfabricated neural probe. Furthermore, our self-assembled optrode was capable of performing a multifunction of electrical/optical stimulation and concurrent recordings of LFPs and Ca^2+^ fluorescence signals, which was used to investigate the relationship between Ca^2+^ signaling and electrophysiological performance. In addition, the simulated data for different intensities of DBS, including estimated evoked VTA volumes and corresponding changes of MC-simulated Ca^2+^ fluorescence signals, were confirmed with a strongly positive correlation to in vivo recordings using our self-assembled optrode. These data suggest that the performance of electrophysiology was consistent with the phenomenon of Ca^2+^ influx to neural synapses, corresponding to the role of Ca^2+^ as a neurotransmitter mediator and subsequently inducing postsynaptic potentials. The developed optrode was successfully used to validate our numerical results on the DBS-evoked VTA estimation and corresponding Ca^2+^ fluorescence excitation from the Monte Carlo modeling. It therefore is a promising tool for the investigation of the coupling between electrophysiology and cellular Ca^2+^ signals in the neuroscience research field.

## Data Availability

The datasets generated for this study are available on request to the corresponding author.

## References

[1] McCormick D.A., Wang Z., Huguenard J. (1993). Neurotransmitter Control of Neocortical Neuronal Activity and Excitability. Cereb. Cortex.

[2] Brady S.T., Lasek R.J., Allen R.D. (1982). Fast Axonal Transport in Extruded Axoplasm from Squid Giant Axon. Science.

[3] Köfalvi A., Rodrigues R.J., Ledent C., Mackie K., Vizi E.S., Cunha R.A., Sperlágh B. (2005). Involvement of Cannabinoid Receptors in the Regulation of Neurotransmitter Release in the Rodent Striatum: A Combined Immunochemical and Pharmacological Analysis. J. Neurosci..

[4] Calia A.B., Masvidal-Codina E., Smith T.M., Schäfer N., Rathore D., Rodríguez-Lucas E., Illa X., De la Cruz J.M., Del Corro E., Prats-Alfonso E. (2022). Full-bandwidth electrophysiology of seizures and epileptiform activity enabled by flexible graphene microtransistor depth neural probes. Nat. Nanotechnol..

[5] Sassenhagen J. (2019). How to analyse electrophysiological responses to naturalistic language with time-resolved multiple regression. Lang. Cogn. Neurosci..

[6] Yin P., Liu Y., Xiao L., Zhang C. (2021). Advanced Metallic and Polymeric Coatings for Neural Interfacing: Structures, Properties and Tissue Responses. Polymers.

[7] Woeppel K.M., Cui X.T. (2021). Nanoparticle and Biomolecule Surface Modification Synergistically Increases Neural Electrode Recording Yield and Minimizes Inflammatory Host Response. Adv. Healthc. Mater..

[8] Buijink A., Piña-Fuentes D., Stam M., Bot M., Schuurman P., Munckhof P.V.D., van Rootselaar A., de Bie R., Beudel M. (2022). Thalamic local field potentials recorded using the deep brain stimulation pulse generator. Clin. Neurophysiol. Pract..

[9] Wang X., Wang M., Sheng H., Zhu L., Zhu J., Zhang H., Liu Y., Zhan L., Wang X., Zhang J. (2022). Subdural neural interfaces for long-term electrical recording, optical microscopy and magnetic resonance imaging. Biomaterials.

[10] Zhang W., Sun C., Shao Y., Zhou Z., Hou Y., Li A. (2019). Partial depletion of dopaminergic neurons in the substantia nigra impairs olfaction and alters neural activity in the olfactory bulb. Sci. Rep..

[11] Tan L.L., Oswald M.J., Heinl C., Romero O.A.R., Kaushalya S.K., Monyer H., Kuner R. (2019). Gamma oscillations in somatosensory cortex recruit prefrontal and descending serotonergic pathways in aversion and nociception. Nat. Commun..

[12] Zhang Z., Constandinou T.G. (2021). Adaptive spike detection and hardware optimization towards autonomous, high-channel-count BMIs. J. Neurosci. Methods.

[13] Cummins D.D., Kochanski R.B., Gilron R., Swann N.C., Little S., Hammer L.H., Starr P.A. (2021). Chronic Sensing of Subthalamic Local Field Potentials: Comparison of First and Second Generation Implantable Bidirectional Systems Within a Single Subject. Front. Neurosci..

[14] Kluger D.S., Balestrieri E., A Busch N., Gross J. (2021). Respiration aligns perception with neural excitability. Elife.

[15] Esghaei M., Daliri M.R., Treue S. (2018). Attention decouples action potentials from the phase of local field potentials in macaque visual cortical area MT. BMC Biol..

[16] Xu G., Wang N., Guo M., Zhang T., Tong Y. (2020). Analysis of time-frequency characteristics and coherence of local field potentials during working memory task of rats after high-frequency repeated transcranial magnetic stimulation. J. Biomed. Eng..

[17] Eslami M., Sadeghi B., Goshadrou F. (2018). Chronic ghrelin administration restores hippocampal long-term potentiation and ameliorates memory impairment in rat model of Alzheimer’s disease. Hippocampus.

[18] Smart O., Choi K.S., Riva-Posse P., Tiruvadi V., Rajendra J., Waters A.C., Crowell A.L., Edwards J., Gross R.E., Mayberg H.S. (2018). Initial Unilateral Exposure to Deep Brain Stimulation in Treatment-Resistant Depression Patients Alters Spectral Power in the Subcallosal Cingulate. Front. Comput. Neurosci..

[19] Telkes I., Viswanathan A., Jimenez-Shahed J., Abosch A., Ozturk M., Gupte A., Jankovic J., Ince N.F. (2018). Local field potentials of subthalamic nucleus contain electrophysiological footprints of motor subtypes of Parkinson’s disease. Proc. Natl. Acad. Sci. USA.

[20] Lipstein N., Chang S., Lin K.-H., López-Murcia F.J., Neher E., Taschenberger H., Brose N. (2021). Munc13-1 is a Ca2+-phospholipid-dependent vesicle priming hub that shapes synaptic short-term plasticity and enables sustained neurotransmission. Neuron.

[21] Hashimoto T., Elder C.M., Vitek J.L. (2001). A template subtraction method for stimulus artifact removal in high-frequency deep brain stimulation. J. Neurosci. Methods.

[22] Shimomura O., Musicki B., Kishi Y., Inouye S. (1993). Light-emitting properties of recombinant semisynthetic aequorins and recombinant fluorescein-conjugated aequorin for measuring cellular calcium. Cell Calcium.

[23] Wang Q., Kong Y., Wu D.-Y., Liu J.-H., Jie W., You Q.-L., Huang L., Hu J., Chu H.-D., Gao F. (2021). Impaired calcium signaling in astrocytes modulates autism spectrum disorder-like behaviors in mice. Nat. Commun..

[24] Bancroft E.A., Srinivasan R. (2021). Emerging Roles for Aberrant Astrocytic Calcium Signals in Parkinson’s Disease. Front. Physiol..

[25] Cho A.-N., Bright F., Morey N., Au C., Ittner L.M., Ke Y.D. (2022). Efficient Gene Expression in Human Stem Cell Derived-Cortical Organoids Using Adeno Associated Virus. Cells.

[26] Kaszas A., Szalay G., Slézia A., Bojdán A., Vanzetta I., Hangya B., Rózsa B., O’Connor R., Moreau D. (2021). Two-photon GCaMP6f imaging of infrared neural stimulation evoked calcium signals in mouse cortical neurons in vivo. Sci. Rep..

[27] Cao H., Gu L., Mohanty S.K., Chiao J.-C. (2013). An Integrated μLED Optrode for Optogenetic Stimulation and Electrical Recording. IEEE Trans. Biomed. Eng..

[28] Wang M., Gu X.-W., Ji B.-W., Wang L.-C., Guo Z.-J., Yang B., Wang X.-L., Li C.-Y., Liu J.-Q. (2019). Three-dimensional drivable optrode array for high-resolution neural stimulations and recordings in multiple brain regions. Biosens. Bioelectron..

[29] Wu F., Stark E., Im M., Cho I.-J., Yoon E.-S., Buzsáki G., Wise K.D., Yoon E. (2013). An implantable neural probe with monolithically integrated dielectric waveguide and recording electrodes for optogenetics applications. J. Neural Eng..

[30] Huby N., Vié V., Renault A., Beaufils S., Lefèvre T., Paquet-Mercier F., Pézolet M., Bêche B. (2013). Native spider silk as a biological optical fiber. Appl. Phys. Lett..

[31] Vázquez G., Valiente R., Gómez-Salces S., Flores-Romero E., Rickards J., Trejo-Luna R. (2016). Carbon implanted waveguides in soda lime glass doped with Yb 3+ and Er 3+ for visible light emission. Opt. Laser Technol..

[32] Son Y., Lee H.J., Kim J., Shin H., Choi N., Lee C.J., Yoon E.-S., Yoon E., Wise K.D., Kim T.G. (2015). In vivo optical modulation of neural signals using monolithically integrated two-dimensional neural probe arrays. Sci. Rep..

[33] LeChasseur Y., Dufour S., Lavertu G., Bories C., Deschênes M., Vallée R., De Koninck Y. (2011). A microprobe for parallel optical and electrical recordings from single neurons in vivo. Nat. Methods.

[34] Grossman N., Poher V., Grubb M.S., Kennedy G.T., Nikolic K., McGovern B., Palmini R.B., Gong Z., Drakakis E.M., A A Neil M. (2010). Multi-site optical excitation using ChR2 and micro-LED array. J. Neural Eng..

[35] McAlinden N., Massoubre D., Richardson E., Gu E., Sakata S., Dawson M.D., Mathieson K. (2013). Thermal and optical characterization of micro-LED probes for in vivo optogenetic neural stimulation. Opt. Lett..

[36] Humar M., Kwok S.J., Choi M., Yetisen A.K., Cho S., Yun S.-H. (2017). Toward biomaterial-based implantable photonic devices. Nanophotonics.

[37] Ji B., Guo Z., Wang M., Yang B., Wang X., Li W., Liu J. (2018). Flexible polyimide-based hybrid opto-electric neural interface with 16 channels of micro-LEDs and electrodes. Microsystems Nanoeng..

[38] Xie X., Chorsi H.T., Agashiwala K., Chang H.M., Kang J., Chu J.H., Sarpkaya I., Htoon H., Schuller J.A., Banerjee K. (2021). The scaling of the microLED and the advantage of 2D materials. arXiv.

[39] Lee H.E. (2021). Novel Bio-Optoelectronics Enabled by Flexible Micro Light-Emitting Diodes. Electronics.

[40] Gutruf P., A Rogers J. (2018). Implantable, wireless device platforms for neuroscience research. Curr. Opin. Neurobiol..

[41] Krioukov E., Greve J., Otto C. (2003). Performance of integrated optical microcavities for refractive index and fluorescence sensing. Sensors Actuators B Chem..

[42] Gómez-Arribas L.N., Benito-Peña E., Hurtado-Sánchez M.D.C., Moreno-Bondi M.C. (2018). Biosensing Based on Nanoparticles for Food Allergens Detection. Sensors.

[43] Sagarzazu G., Bedu M., Martinelli L., Pelletier N., Safarov V.I., Weisbuch C., Gacoin T., Benisty H. (2009). Quantitative analysis of enhanced light irradiance in waveguide-based fluorescent microarrays. Biosens. Bioelectron..

[44] Schor J.S., Montalvo I.G., Spratt P.W., Brakaj R.J., A Stansil J., Twedell E.L., Bender K.J., Nelson A.B., Program N., University of California (2022). Therapeutic deep brain stimulation disrupts movement-related subthalamic nucleus activity in parkinsonian mice. Elife.

[45] Sun Q., Zhang J., Li A., Yao M., Liu G., Chen S., Luo Y., Wang Z., Gong H., Li X. (2022). Acetylcholine deficiency disrupts extratelencephalic projection neurons in the prefrontal cortex in a mouse model of Alzheimer’s disease. Nat. Commun..

[46] Thenaisie Y., Palmisano C., Canessa A., Keulen B.J., Capetian P., Jiménez M.C., Bally J.F., Manferlotti E., Beccaria L., Zutt R. (2021). Towards adaptive deep brain stimulation: Clinical and technical notes on a novel commercial device for chronic brain sensing. J. Neural Eng..

[47] Duffley G., Lutz B.J., Szabo A., Wright A., Hess C.W., Ramirez-Zamora A., Zeilman P., Chiu S., Foote K.D., Okun M.S. (2021). Home health management of Parkinson disease deep brain stimulation: A randomized clinical trial. J. Am. Med. Assoc. Neurol..

[48] Wu H., Kakusa B., Neuner S., Christoffel D.J., Heifets B.D., Malenka R.C., Halpern C.H. (2022). Local accumbens in vivo imaging during deep brain stimulation reveals a strategy-dependent amelioration of hedonic feeding. Proc. Natl. Acad. Sci. USA.

[49] Jakobs M., Fomenko A., Lozano A., Kiening K.L. (2019). Cellular, molecular, and clinical mechanisms of action of deep brain stimulation—A systematic review on established indications and outlook on future developments. EMBO Mol. Med..

[50] Akram H., Sotiropoulos S.N., Jbabdi S., Georgiev D., Mahlknecht P., Hyam J., Foltynie T., Limousin P., De Vita E., Jahanshahi M. (2017). Subthalamic deep brain stimulation sweet spots and hyperdirect cortical connectivity in Parkinson’s disease. Neuroimage.

[51] Horn A., Li N., Dembek T.A., Kappel A., Boulay C., Ewert S., Tietze A., Husch A., Perera T., Neumann W.-J. (2018). Lead-DBS v2: Towards a comprehensive pipeline for deep brain stimulation imaging. Neuroimage.

[52] Noecker A.M., Choi K.S., Riva-Posse P., Gross R.E., Mayberg H.S., McIntyre C.C. (2018). StimVision Software: Examples and Applications in Subcallosal Cingulate Deep Brain Stimulation for Depression. Neuromodul. Technol. Neural Interface.

[53] Fritz N., Gulick D., Bailly M., Christen J.M.B. (2021). Modeling optical design parameters for fine stimulation in sciatic nerve of optogenetic mice. Sci. Rep..

[54] Keck C.H.C., Rommelfanger N.J., Ou Z., Hong G. (2021). Bioinspired nanoantennas for opsin sensitization in optogenetic applications: A theoretical investigation. Multifunct. Mater..

[55] Zhu C., Liu Q. (2013). Review of Monte Carlo modeling of light transport in tissues. J. Biomed. Opt..

[56] Thiry D., Molina-Luna L., Gautron E., Stephant N., Chauvin A., Du K., Ding J., Choi C.-H., Tessier P.-Y., El Mel A.-A. (2015). The Kirkendall Effect in Binary Alloys: Trapping Gold in Copper Oxide Nanoshells. Chem. Mater..

[57] Gan C.L., Classe F.C., Chan B.L., Hashim U. (2014). Evolution and investigation of copper and gold ball bonds in extended reliability stressing. Gold Bull..

[58] Gan C.L., Hashim U. (2013). Reliability Assessment and Activation Energy Study of Au and Pd-Coated Cu Wires Post High Temperature Aging in Nanoscale Semiconductor Packaging. J. Electron. Packag..

[59] Ravi R., Paul A. (2012). Diffusion mechanism in the gold-copper system. J. Mater. Sci. Mater. Electron..

[60] Zharkov S.M., Moiseenko E.T., Altunin R.R. (2019). L10 ordered phase formation at solid state reactions in Cu/Au and Fe/Pd thin films. J. Solid State Chem..

[61] Lai H.-Y., Younce J.R., Albaugh D.L., Kao Y.-C.J., Shih Y.-Y.I. (2014). Functional MRI reveals frequency-dependent responses during deep brain stimulation at the subthalamic nucleus or internal globus pallidus. Neuroimage.

[62] Butson C.R., McIntyre C.C. (2005). Role of electrode design on the volume of tissue activated during deep brain stimulation. J. Neural Eng..

[63] Amendola C., Spinelli L., Contini D., De Carli A., Martinelli C., Fumagalli M., Torricelli A. (2021). Accuracy of homogeneous models for photon diffusion in estimating neonatal cerebral hemodynamics by TD-NIRS. Biomed. Opt. Express.

[64] Chang K.-T., Lin Y.-Y., Lin Y.-Y., Lin Y.-L., Cheng H., Chang Y., Huang M.-C. (2019). In Vivo Real-Time Discrimination Among Glioma, Infiltration Zone, and Normal Brain Tissue via Autofluorescence Technology. World Neurosurg..

[65] Ung K., Arenkiel B. (2012). Fiber-optic implantation for chronic optogenetic stimulation of brain tissue. J. Vis. Exp..

[66] Koss A.M., Alterman R.L., Tagliati M., Shils J.L. (2005). Calculating total electrical energy delivered by deep brain stimulation systems. Ann. Neurol..

[67] Marks W.J., Ostrem J.L. (2015). Deep Brain Stimulation Management.

[68] Yang P.-F., Chen Y.-Y., Chen D.-Y., Hu J.W., Chen J.-H., Yen C.-T. (2013). Comparison of fMRI BOLD Response Patterns by Electrical Stimulation of the Ventroposterior Complex and Medial Thalamus of the Rat. PLoS ONE.

[69] Siegle J.H., Lopez A.C., Patel Y.A., Abramov K., Ohayon S., Voigts J. (2017). Open Ephys: An open-source, plugin-based platform for multichannel electrophysiology. J. Neural Eng..

[70] Zhou H., Neville K.R., Goldstein N., Kabu S., Kausar N., Ye R., Nguyen T.T., Gelwan N., Hyman B.T., Gomperts S.N. (2019). Cholinergic modulation of hippocampal calcium activity across the sleep-wake cycle. Elife.

[71] Stocke S.K., Samuelsen C.L. (2020). A drivable optrode for use in chronic electrophysiology and optogenetic experiments. J. Neurosci. Methods.

[72] Sileo L., Bitzenhofer S.H., Spagnolo B., Pöpplau J.A., Holzhammer T., Pisanello M., Pisano F., Bellistri E., Maglie E., De Vittorio M. (2018). Tapered Fibers Combined With a Multi-Electrode Array for Optogenetics in Mouse Medial Prefrontal Cortex. Front. Neurosci..

[73] Pisanello F., Mandelbaum G., Pisanello M., Oldenburg I.A., Sileo L., Markowitz J.E., Peterson R.E., Della Patria A., Haynes T.M., Emara M.S. (2017). Dynamic illumination of spatially restricted or large brain volumes via a single tapered optical fiber. Nat. Neurosci..

[74] Butson C.R., Cooper S.E., Henderson J.M., McIntyre C.C. (2007). Patient-specific analysis of the volume of tissue activated during deep brain stimulation. Neuroimage.

[75] Paulk A.C., Zelmann R., Crocker B., Widge A.S., Dougherty D.D., Eskandar E.N., Weisholtz D.S., Richardson R.M., Cosgrove G.R., Williams Z.M. (2022). Local and distant cortical responses to single pulse intracranial stimulation in the human brain are differentially modulated by specific stimulation parameters. Brain Stimul..

[76] Chang Y.-C., Walston S.T., Chow R.H., Weiland J.D. (2017). GCaMP expression in retinal ganglion cells characterized using a low-cost fundus imaging system. J. Neural Eng..

[77] Michelson N.J., Vanni M.P., Murphy T.H. (2019). Comparison between transgenic and AAV-PHP. eB-mediated expression of GCaMP6s using in vivo wide-field functional imaging of brain activity. Neurophotonics.

[78] Kawata S., Mukai Y., Nishimura Y., Takahashi T., Saitoh N. (2022). Green fluorescent cAMP indicator of high speed and specificity suitable for neuronal live-cell imaging. Proc. Natl. Acad. Sci. USA.

[79] Park J.E., Zhang X.F., Choi S.-H., Okahara J., Sasaki E., Silva A.C. (2016). Generation of transgenic marmosets expressing genetically encoded calcium indicators. Sci. Rep..

[80] Kessi M., Chen B., Peng J., Yan F., Yang L., Yin F. (2021). Calcium channelopathies and intellectual disability: A systematic review. Orphanet J. Rare Dis..

[81] Eshra A., Schmidt H., Eilers J., Hallermann S. (2021). Calc ium dependence of neurotransmitter release at a high fidelity synapse. Elife.

[82] Trevathan J.K., Asp A.J., Nicolai E.N., Trevathan J.M., Kremer N.A., Kozai T.D., Cheng D., Schachter M.J., Nassi J.J., Otte S.L. (2021). Calcium imaging in freely moving mice during electrical stimulation of deep brain structures. J. Neural Eng..

[83] Sych Y., Chernysheva M., Sumanovski L.T., Helmchen F. (2019). High-density multi-fiber photometry for studying large-scale brain circuit dynamics. Nat. Methods.

[84] Hammer L.H., Kochanski R.B., Starr P.A., Little S. (2022). Artifact Characterization and a Multipurpose Template-Based Offline Removal Solution for a Sensing-Enabled Deep Brain Stimulation Device. Ster. Funct. Neurosurg..

[85] Chen T.-W., Wardill T.J., Sun Y., Pulver S.R., Renninger S.L., Baohan A., Schreiter E.R., Kerr R.A., Orger M.B., Jayaraman V. (2013). Ultrasensitive fluorescent proteins for imaging neuronal activity. Nature.

[86] Legaria A.A., Matikainen-Ankney B.A., Ben Yang B., Ahanonu B., Licholai J.A., Parker J.G., Kravitz A.V. (2022). Fiber photometry in striatum reflects primarily nonsomatic changes in calcium. Nat. Neurosci..

[87] Cui Y., Huang X., Huang P., Huang L., Feng Z., Xiang X., Chen X., Li A., Ren C., Li H. (2022). Reward ameliorates depressive-like behaviors via inhibition of the substantia innominata to the lateral habenula projection. Sci. Adv..

